# Extracellular vesicles and immunogenic stress in cancer

**DOI:** 10.1038/s41419-021-04171-z

**Published:** 2021-10-01

**Authors:** Qi Wu, Hanpu Zhang, Si Sun, Lijun Wang, Shengrong Sun

**Affiliations:** 1 Department of Breast and Thyroid Surgery, Renmin Hospital of Wuhan University, Wuhan, Hubei P. R. China; 2 Department of Colorectal Surgery, The First Affiliated Hospital, Zhejiang University, Hangzhou, China; 3 Department of Clinical Laboratory, Renmin Hospital of Wuhan University, Wuhan, Hubei P. R. China

**Keywords:** Immunosurveillance, Autophagy, Cancer

## Abstract

Tumor progression requires bidirectional cell-to-cell communication within a complex tumor microenvironment (TME). Extracellular vesicles (EVs) as carriers have the capacity to shuttle regulatory molecules, including nucleic acids, proteins, and lipids, between cancer cells and multiple stromal cells, inducing remarkable phenotypic alterations in the TME. Recently proposed the concept “immunogenic stress”, which means in some stressed microenvironment, cancer cells can release EVs containing specific immunoregulatory mediators, depending on the initiating stress-associated pathway, thereby provoking the changes of immune status in the TME. Considerable evidence has revealed that the intracellular mechanisms underlying the response to diverse stresses are mainly autophagy, endoplasmic reticulum (ER) stress reactions and the DNA damage response (DDR). In addition, the activation of immunogenic stress responses endows hosts with immune surveillance capacity; in contrast, several cargoes in EVs under immunogenic stress trigger a passive immune response by mediating the function of immune cells. This review discusses the current understanding of the immunogenic stress pathways in cancer and describes the interrelation between EVs and immunogenic stress to propose potential treatment strategies and biomarkers.

## Facts

- Immunogenic stress (IS) is defined as a cell stress modality which stimulates an immune response against stress-cell antigens, particularly cancer cell antigens.
- Immunogenic stress (IS) including autophagy, ER stress reactions and the DNA damage response, selectively encapsulates complex components including various RNAs, proteins, and lipids into extracellular vesicles, that mediate crosstalk between immune cells in tumor microenvironment.
- The EVs-targeted reactions elicited by cellular stress have major implications for anticancer treatment.

## Questions

- What is the mechanism by which specific immunogenic proteins, DNA, and RNA are selectively packaged into extracellular vesicles (EVs)?
- How do EVs-containing specific mediators from stressful malignant cells orchestrate the reconstruction of tumor microenvironment?

## Introduction

The tumor microenvironment (TME) is a complex system comprising cancer cells and multiple stromal cells, including different subtypes of immune cells. To maintain cellular homeostasis and escape host anticancer immunity, cancer cells have developed intercellular communication pathways, such as the secretion of mediators. In addition, the extracellular vesicles (EVs), as a new paradigm, has emerged as a mechanism of intercellular communication [1]. EVs are involved in various normal physiological processes, including immune modulation [2], cell differentiation [3], and tissue repair and regeneration [4].

EVs are regarded as membrane vesicles with heterogeneous profiles, and there are at least three major categories of EVs: apoptotic bodies, exosomes, and microvesicles (MVs) [5]. Once secreted in the extracellular space, EVs can be absorbed by recipient cells. Diverse messengers, including DNA, RNA, proteins, and lipids, can be contained in EVs through which they are trafficked between cells to promote cell-to-cell communication at the paracrine and circulation levels. Moreover, all types of tumor cells can secrete EVs, which have been detected in several bodily fluids, such as blood, bile, urine, and breast milk [6]. EVs also participate in a wide range of pathological processes in the progression of cancer and are increasingly recognized as hallmarks of cancer [7]. Furthermore, emerging data from studies have indicated that several kinds of cell stress, including DNA damage stress, autophagy, and endoplasmic reticulum (ER) stress, can stimulate an increase in the number of EVs released, thus creating a distinct type of damage-associated molecular patterns (DAMPs) that shape immune responses [8]. An understanding of how cancer cells under stress conditions utilize EVs as an approach to mediate immune reactions may contribute to the use of EVs as novel biomarkers or to the manipulation of them for use in therapeutic applications.

Cells undergoing malignant growth and metastasis require frequently inducible adaptive capacity despite multiple cell-intrinsic and cell-extrinsic challenges. Notably, the concept of immunogenic stress (IS) has emerged, suggesting that a cell stress modality stimulates an immune response against stress-cell antigens, particularly cancer cell antigens [9]. IS is the central inducer in an extensive network of fundamental pathways, including those of autophagy, ER stress reactions, and the DNA damage response (DDR), that regulate adaptive capacity. Under immunogenic stress, cancer cells can selectively release immunogenic transmitters into the microenvironment to trigger an immune response. Furthermore, the capacity to tolerate IS enables cells to stimulate immunosuppression factors. Therefore, it is crucial to balance the stress state in cancer cells and to discover the features and functions of the stress-specific cargoes transported during the immune response.

The apparent mechanisms that connect cellular stress reactions to paracrine pathways and contribute to sustained immune homeostasis have been discovered. This cell-to-cell interplay is achieved via multiple signals and can alter the microenvironment of stressed cancer cells, and the active or passive secretion of EVs containing several specific proteins, RNA, and/or DNA. In this review, we discuss the intertwined association of EVs and immunogenic stress, particularly how different cell stress patterns impact the biogenesis and properties of the EVs in the TME. Furthermore, we describe the immunogenic roles of their cargoes, including oncoproteins, RNA species, and DNA fragments. We pay particular attention to the modalities of stressed-associated EVs in therapeutic responses and predictors. Together, the association of EVs and immunogenic stress in cancer has important implications for immunotherapy strategies used to fight cancer, making EVs good prospects for clinical application.

## The synthesis and release of EVs and cell stress: a complex relationship

Indeed, the composition of EVs depends on their cellular origin and cell status. The secretion profile of cells changes profoundly in response to intercellular stress, which is mainly induced by autophagy, endoplasmic reticulum stress, or damaged DNA. In the following sections, the relationships among the main stress reactions, such as autophagy, ER stress reactions and DDR, and EVs are described.

### EVs and autophagy

Autophagy is an evolutionarily conserved process among almost all eukaryotes in which organelles and intercellular proteins are sequestrated by autophagosomes and degraded when the autophagosomes fuse with lysosomes [10]. In addition to the canonical degradation function, the autophagy machinery also contributes to the secretion of intercellular proteins. Emerging data have indicated that autophagy direct links to the biogenesis of exosomes via shared organelles or molecular pathways.

**Fig. 1:**
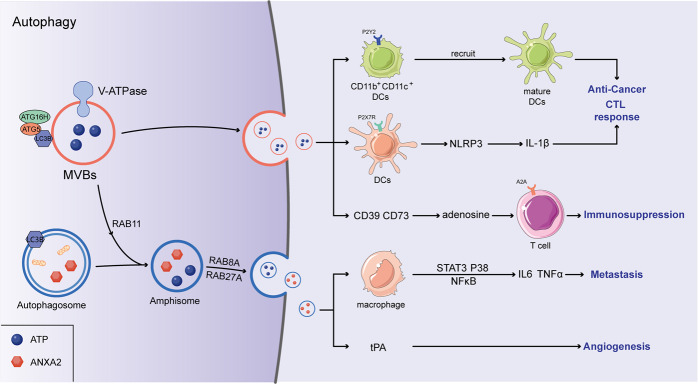
Autophagy and the release of immune-related EVs. The autophagy-associated complex (ATG5–ATG16) translocalized to MVBs and dissociated vacuolar proton pumps (V-ATPase) from MVBs, in which the acidification of the MVBs lumen was inhibited. Ultimately, the secretion of EVs augmented via MVBs–plasma membrane (PM) fusion. In addition, amphisomes were formed by the fusion of autophagosomes and MVBs, which accelerated the EVs release controlled by RAB8A and RAB27A. Immunoregulatory factors including ANXA2 and ATP were contained in EVs, ANXA2 can induce an increased secretion of TNFα and IL6 by activating the STAT3, p38, and NF-κB pathways in macrophages. In addition, ANXA2 promotes tPA-dependent angiogenesis. ATP can promote the differentiation of CD11b^+^CD11c^+^Ly6C^high^ cells into mature DCs by binding with purinergic receptor P2Y2, and upregulate the IL-1β production through NLRP3 inflammasome, ultimately stimulating the activation of anti-tumor CTL responses. However, adenosine can be converted from ATP hydrolyzed by CD39 and CD73, further inhibiting T-cell activation through binding with the adenosine A(2 A) receptor.

The interactions between autophagy and exosome biogenesis through diverse pathways has gained increasing interest (Fig. 1). At the molecular level, the role of autophagy-associated proteins in exosome biogenesis has been revealed. For example, recent studies have shown that autophagy-related 5 (ATG5) and ATG16L1 are potential regulators of exosome biogenesis [11]. Specifically, the ATG5–ATG16 complex localized to MVBs can mediate the lipidation of LC3B (a marker of autophagic flux) and promote MVB–plasma membrane (PM) fusion and exosome release by dissociating vacuolar proton pumps (V1Vo-ATPase) from MVBs and inhibiting the acidification of the MVB lumen. Moreover, the ATG12–ATG3 complex has also been demonstrated to regulate the biogenesis of exosomes by interacting with ALG-2-interacting protein X (ALIX), an ESCRT-associated protein pivotal to exosome biogenesis [12]. Specifically, cells lacking ATG12–ATG3-controlled MVB morphology, accumulate perinuclear late endosomes and have reduced exosome biogenesis. Recent research described a new autophagic secretion pathway called LC3-conjugation pathway, in which multitudinous RNA-binding proteins (RBPs) and small non-coding RNAs were packaged into EVs and secreted via EVs. Scaffold-attachment factor B (SAFB) and heterogeneous nuclear ribonucleoprotein K (HNRNPK), two representative RBPs, were demonstrated to interact with LC3 and were excreted through lipidated LC3-enriched EVs [13]. Overall, this evidence demonstrates that the core proteins in autophagy can directly mediate the fate of MVBs, thereby affecting the biogenesis of exosomes. At the organelle level, amphisomes formed by MVBs fusing with autophagosomes have multiple fates, including lysosomal degradation and extracellular release [14]. Importantly, amphisome degradation suggests antagonism between exosome release and autophagy. In erythroleukemic cells, rapamycin treatment or starvation can increase autophagosome–MVB fusion and decrease exosomes release through an unclear mechanism [15]. Similarly, a reduced release of exosomes can promote the redirection of MVBs into the autophagic degradation pathway. In vitro and in vivo, ISGylation, the conjugation of the ubiquitin-like protein ISG15, has been consistently demonstrated to promote protein degradation accompanied by decreased MVBs and exosome release [16]. Furthermore, the inhibition of endosome–lysosome fusion or inhibition of autophagy through the administration of bafilomycin A1, a dominant-negative mutant form of RAB7, can rescue exosome release, supporting the notion that autophagy participates in the degradation of MVBs, which contain ISGylation-induced aggregates [16, 17]. The evidence seems to indicate that autophagy-dependent degradation of MVBs prevails in diverse contexts. Notably, amphisomes can also release their contents after they fuse with the plasma membrane. In addition, it has been reported that, constitutively active IKKβ subunits (CA-IKKβ) take part in the interactions between autophagy and endosomal system. CA-IKKβ induces accumulation of autophagosomes and their fusion with MVBs to form amphisomes in cancer cells, and also drives the release of EVs containing autophagy components through an amphisome-dependent mechanism. Indicated that, under stress conditions, the coordinated action of the autophagy and endosomal systems in tumor cells is essential for maintaining cellular homeostasis and survival [18]. Thus, the connection between autophagy and EVs is complex and needs further work to elucidate the mechanism by which autophagy regulates EV secretion. Interestingly, unconventional roles of autophagy have begun to emerge: autophagy-dependent exosomes, as pivotal messengers in intercellular communication, participate in the interplay between tumor cells, immune cells, and other stromal cells [19]. Thus, it would be very interesting to explore whether autophagic secretion can be regulated by exosome-mediated signal transduction.

It has become clear that the interplay between autophagic processes and exosome biogenesis has important implications. However, despite the roles of exosome signaling and autophagy in cancer cells that have been individually reviewed, the significance of the association between autophagy and exosomes in cancer is only now beginning to be recognized. In prostate and breast cancer cell lines, mitochondrial damage induced by rotenone contributes to an increase in the levels of ATG7 and endosomal tetraspanins in the cytoplasm, which are associated with increased autophagy and exosome release [20]. Moreover, in pancreatic cancer cells, the GAIP-interacting protein C-terminus (GIPC), a regulatory protein of vesicular trafficking signaling pathways, has been found to simultaneously regulate autophagy and exosome biogenesis. GIPC depletion may lead to increased autophagic flux and exosome production by decreasing mTOR activity [21]. Additionally, cancer cells may have a mechanism of maintaining homeostasis by redirecting cargo destined to degradation to exosomal secretion. For example, it has been found that FYVE-type zinc finger-containing phosphoinositide kinase (PIKfyve) can redirect proteins from the pathway to autophagic degradation to that of exosomal secretion in prostate cancer cells [22]. And the inhibition or downregulation of PIKfyve with apilimod or siRNA can prevent autophagic flux but increase the secretion of EVs expressing canonical exosome biomarkers such as TSG101 and ALIX, as well as a subclass of ATG [22]. In addition, recent study reported picropodophyllin (PPP) and linsitinib as an inhibitor of the IGF1R could significantly enhance the autophagic flux and the infiltration of cytotoxic T lymphocytes in tumor combined with chemotherapy, revealing that autophagy-induced immune response may play a key role in anti-cancer treatment [23, 24]. Moreover, the release of autophagy-related EVs could promote the phenotype of cancer steam cell in glioma and breast cancer cells, thereby enabling them to survive in harsh conditions such as hypoxia or nutritional deficiencies [25]. Therefore, it is imperative to further investigate the mechanism by which autophagy and exosome secretion interrelate to enhance the stress adaptation of cancer cells.

### EVs and ER stress

The endoplasmic reticulum, as a protein factory, plays a key role in maintaining cellular homeostasis. The folding and posttranslational modification of membranes and secretory proteins are performed in the ER. The disruption of ER homeostasis, known as ER stress, can be induced by various environmental and genetic factors. Under ER stress conditions, the unfolded protein response (UPR) is initiated by three ER stress transducers: protein kinase R-like ER kinase (PERK), inositol required enzyme 1α (IRE1α), and activating transcription factor 6 (ATF6) [26]. UPR activation can maintain ER homeostasis by promoting the production of ER folding machinery proteins and the degradation of misfolded proteins. Thus, it is conceivable that ER stress is closely associated with the production and secretion of EVs.

**Fig. 2:**
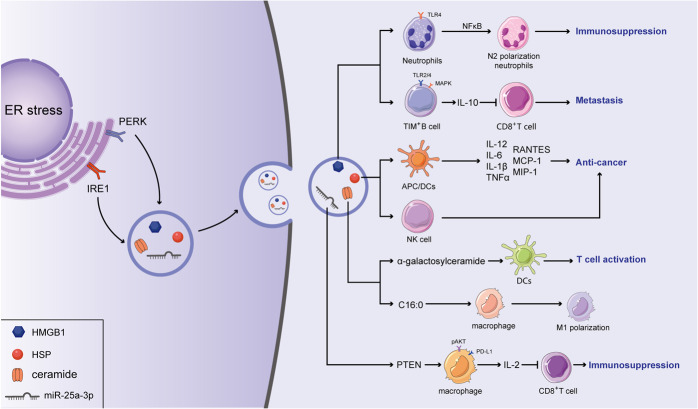
ER stress regulate the secretion of EVs which contained immunomodulatory factors. In response to ER stress, ER stress transducers (IRE1 and PERK) facilitated the MVBs formation and boosted the EVs-mediated release of HMGB1, HPSs, ceramide, and miRNA. And HMGB1 has been reported to suppressed anti-tumor immune response by promoting the N2 polarization of neutrophils via activating the NF-κB pathway. Moreover, HMGB1 has an inhibitory effect on CD8^+^ T cells by stimulating the expansion of the TIM-1+Breg cells and increasing the expression of IL-10 through the MAPK and TLR 2/4 pathways. Exosomal HSP90, HSP70, and HSP60 have been indicated to promote the chemotherapy resistance of cancer cells. Nevertheless, Hsp72 activates the effect of NK cells, simultaneously promoting the release of inflammatory factors in APCs to stimulate the anti-cancer response. C24:1 and C18:0 ceramides promote chemoresistance in multiple cancer while alpha-galactosylceramide enhances T-cell-mediated anti-cancer response through the activated effect of dendritic cells. miR-23a-3p decreases the production of IL-2 and recedes the function of CD8^+^ T cells by inhibiting PTEN and then increasing the AKT-mediated PD-L1 expression in macrophages.

Recently, an increasing number of studies have indicated the pivotal role of ER stress in EV release, which is associated with disease progression, including cancer progression (Fig. 2). In hepatocytes, ER stress activated by palmitate (PA) can induce a significant increase in EV release through IRE1α/X-box binding protein-1. PA-induced EV secretion reportedly regulated macrophage chemotaxis through sphingosine-1-phosphate (S1P) produced by C16:0 ceramide, which is an important component found in EVs. This potential mechanism is thought to contribute to the development of nonalcoholic steatohepatitis (NASH) by recruiting macrophages to the lipotoxic liver [27]. In terms of cancer cells, it has been demonstrated that ER stress can enhance MVB formation and exosome release through IRE1 and PERK in HeLa cells [28]. Similarly, severe ER stress in choriocarcinoma cells has also been shown to contribute to the release of EVs carrying DAMP molecules, such as high mobility group protein B1 (HMGB1), heat shock protein 70 (hsp70), and/or histone H3 [29]. In addition, hepatocarcinoma Huh-7 cells treated by thapsigargin, a conventional ER stress inducer, shows an increased level of EVs release, proving the key regulatory role of ER stress in the process of tumor cell EVs release [30]. Notably, cancer cells can transmit information on ER stress to other cells in a phenomenon called “transmissible ER stress” (TERS). TERS improve the production of interleukin 6 (IL-6) and chemokines, thereby inducing the resistance of cells to nutrient deprivation and genotoxic stress [31]. Moreover, this transmission can promote macrophage activation in the tumor microenvironment to eventually facilitate tumor progression [32]. Therefore, considering the role of EVs in cancer cachexia, the release of exosomes can possibly play a role in the TERS caused inflammatory and immunosuppressive phenotype of tumors. Together, the data show that the cross-talk among exosomes and ER stress factors may regulate cell behavior and communication with the microenvironment. Further investigations are warranted to interpret the multiple dimensions of exosome–ER interactions.

### EVs and DNA damage stress

**Fig. 3:**
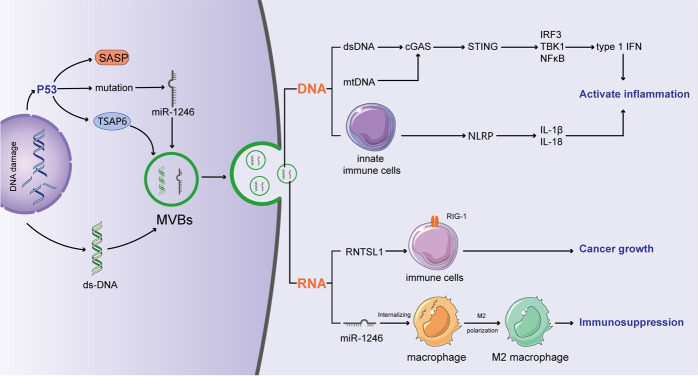
DNA damage stress induced the release of immunomodulatory cargoes through EVs. In DDR, P53 activated the tumor suppressor-activated pathway 6 (TSAP6) to increase the secretion of exosomes. Additionally, mutant P53 can selectively increase the release of miR-1246-enriched exosomes. And these miR-1246-enriched exosomes contribute to anti-inflammatory immunosuppression by inducing high levels of TGF-β activity. As a stress reaction, senescence enhanced the biosynthesis of exosome-like vesicles, and further several highly reactive agents were liberated via EVs. When cellular DNA damages appeared, EVs secretion contributed to maintain intercellular homeostasis via excreting the harmful cytoplasmic DNA fragments. dsDNA and mtDNA can upregulate the expressions of type I IFNs by activating cGAS/STING pathway. Additionally, exoDNA can be absorbed by innate immune cells in the intestine to stimulate the activation of the inflammasome and then enhance the secretion of IL-18 and IL-1β. RN7SL1 activates RIG-I in immune cells to accelerate the tumor growth. Importantly, dsRNA is deemed as a contributor of anti-cancer immunity since it recruits T cells into the tumor microenvironment through increasing the secretion of TLR3-dependent cytokines including type I IFN and CXCL10.

Similar to protein stress, DDR is mainly derived from the accumulation of DNA fragmentation. The prevalence of DDR reflects a number of cancer-associated signaling pathways, the deregulation of which is involved in impairing genomic integrity. DDR can be the result of several mechanisms, such as mitotic replication stress or defects in centrosome replication and oncogenic signaling, and its different forms induce different repair signaling pathways [33], collectively termed the DDR. There are five major repair pathways in human cells that address different types of DNA damage, and different DDRs may compensate in the absence of a specialized repair pathway [34]. Recently, it has become increasingly clear that DDR has a pivotal effect on the composition and secretion of EVs (Fig. 3). It has been indicated that p53, a key DDR factor regulating the G1/S checkpoint, can regulate the secretion of exosomes by activating the tumor suppressor-activated pathway 6 (TSAP6) [35]. Interestingly, colon cancer cells carrying mutp53 have been reported to selectively release miR-1246-enriched exosomes [36]. Likewise, EVs also play a pivotal role in maintaining DNA homeostasis. Human cells can remove harmful cytoplasmic DNA fragments via exosome secretion to maintain cellular homeostasis. When the secretion of exosomes is inhibited, nuclear DNA accumulates in the cytoplasm and activates the cytoplasmic DNA-sensing mechanism, which can activate the innate immune system and thus contribute to ROS-dependent DDR and induce senescence-like growth arrest or apoptosis [37]. These findings, taken together, indicate that EVs and DNA damage stress are highly interrelated through multiple different ways and can regulate the cellular homeostasis and immunity response, which is essential for the development of cancer.

## EV-mediated release of immunomodulators: implications for tumor immunity

The identification of critical immunomodulatory factors in stress-induced EVs has yielded potential information on effective regulators of the tumor immune microenvironment; the immune signaling was listed in Table 1. Moreover, targeting these immunomodulatory factors have potential value in anti-cancer therapy; corresponding drugs were listed in Table 2.

**Table 1:** Regulation of immune responses by immunostimulatory factors contained in EVs.

Immunostimulatory factors contained in EVs	Targeted immune cells	Mechanism of action	Reference
ANXA2	Macrophages	Stimulate the activation of the STAT3, p38, and NF-κB pathways in and the enhanced secretion of TNFα and IL6.	[57]
ATP	CD11b + CD11c + Ly6C high cells	Promote the differentiation of CD11^b+^CD11^c+^Ly6C^high^ cells into mature bona fide DCs.	[66–68]
		Upregulate the productions of IL-1β and stimulate the activation of CTL responses.	[69, 70]
HMGB1	DCs	Promotes the activation of CD4^+^ T cells by stimulating the cross-presentation of cancer cell neoantigens to the immune system by DCs.	[38]
	TIM-1+Breg cells	Stimulated the expansion of the TIM-1+Breg cells through the mitogen-activated protein kinase (MAPK) and Toll-like receptor (TLR) 2/4 pathways.	[39]
Ceramides	DCs	Induce the activations of DCs and thereby enhance the function of T cells	[115]
HSPs	APCs	Increases the release of cytokines, such as IL-12, IL-6, IL-1β, and TNF-α, as well as chemokines, including RANTES, MCP-1, and MIP-1.	[102, 103]
	DCs NK cells	Induce the maturation of DC and NK cell migration by increasing the expression of CD86, CD83, CD40, and MHC class II molecules on the DC surface.	[104–106]
DNA	Immune cells	Stimulate the activation of type I IFN pathway by STING.	[82–87]
RNA	Macrophages	Inhibit the expression of PTEN and increase the expression of phosphorylated AKT and PD-L1 in macrophages.	[90]
	T cells	Decreases the production of IL-2 and the ratio of CD8^+^ T cells but increases the number of apoptotic T cells.	[90]

**Table 2:** Targeting EVs or inoculating engineered exosomes in antitumor therapies.

Target	Drug	Cancer type	Notes	Reference
ANXA2	ch2448	Breast cancer Ovarian cancer	Kill tumor cells by ADCC or ADC routes.	[59, 60]
	Ginsenoside compound K	Astroglial cancer liver cancer	Inhibit the activation of NF-кB.	[61]
CD39	NTPDase	Liver cancer	Stimulate the antitumor immunity and decrease the metastatic of tumor cells.	[72]
CD73	Anti-CD73 antibody	Breast cancer	Inhibit the metastases as well as migration of tumor cell.	[73, 74]
	MEDI9447	Solid tumors	Combine with anti-PD-L1 therapy.	NCT02503774
		Relapsed ovarian cancer	Combine with MEDI0562, Tremelilumab, and Durvalumab.	NCT03267589
HMGB1	HMG Box-A	Mesothelioma	Suppresses the viability, migration, and growth of tumor cells, and decreases the growth of mesothelioma tumors in a xenograft mice model.	[42–44]
	EP	DLBCL Gastric cancer Liver cancer Prostate cancer Gallbladder cancer	Downregulated the expression of HMGB1 and phosphorylation of ERK1/2 and Src.	[45–52]
	Triptolide	Breast cancer	Downregulated the expressions of TLR4 and phosphorylated NF-κB p65.	[53]
	miR-22	Osteosarcoma	Reversion the resistance of tumor cells in chemotherapy by targeting the 3′ UTR of HMGB1.	[54]
Ceramides	Alpha-galactosyl ceramide	Solid tumors	Enhances the activation of T cells by dendritic cells	[75]
HSPs	RP101 Quercetin	Oral cancer Glioblastoma	Enhance the anticancer therapeutic effects in various cell lines including oral cancer and glioblastoma	[107, 108]
	NW457	Conditional reprogramming cells	Induce the apoptosis	[109]
	Ganetespib	Pancreatic cancer	As a radiosensitizer in pancreatic ductal adenocarcinoma by modulating the STAT3, HIF-1α as well as AKT-driven pathways	[110]
DNA	exo-mtDNA	Breast cancer	Treatment with exo-mtDNA could reverse the drug resistance of tumor cells.	[88]
RNA	miR-1246 inhibitor	Ovarian cancer	Combine with chemotherapy led to reduced tumor burden in vivo.	[91]
	PEI/siRNA complexes	Prostate cancer	Inhibit the growth of tumor cells	[92]

### HMGB1

High-mobility group box 1 (HMGB1), a nuclear protein, acts as a DNA chaperone that can reversibly bend DNA by transiently binding with it. HMGB1, identified as a protein of DAMP, normally exists in the nucleus from where it is released upon cell death, endowing the immune system with antigen-recognition ability. Notably, under severe stress, cells actively secrete HMGB1 through a dedicated secretion pathway that relocates HMGB1 to the cytoplasm from the nucleus and then directly to the extracellular space or to secretory lysosomes [38]. Notably, HMGB1 is highly involved in various levels of inflammation and promotes the activation of CD4 T cells by stimulating the cross-presentation of cancer cell neoantigens to the immune system by DCs [39]. Recent studies have shown that HMGB1 can be secreted via exosomes [40, 41]. Regulatory B (Breg) cells are a subset of the B cells that infiltrate solid tumors and show different phenotypes in distinct tumor microenvironments. It was found that a high number of TIM-1+Breg cells had infiltrated into the hepatocellular carcinoma (HCC) tissue in patients compared to the number that had infiltrated into the paired peritumoral tissue. The infiltrating TIM-1+Breg cells exhibiting a CD5highCD24-CD27-/ + CD38 + /high phenotype contributed to high levels of IL-10, which had a strong inhibitory effect on CD8^+^ T cells. Notably, tumor-released exosomes transferred HMGB1 into B cells and then stimulated the expansion of the TIM-1+Breg cells through the mitogen-activated protein kinase (MAPK) and Toll-like receptor (TLR) 2/4 pathways [40]. Moreover, HMGB1 can be released by exosomes derived from gastric cancer cells and then internalized by neutrophils, in which it interacts with TLR4 to activate the NF-κB pathway (Fig. 2). Therefore, tumor-derived exosomes can promote the polarization of neutrophils toward the pro-tumor phenotype, which is a process closely associated with the poor prognosis of gastric cancer patients [42]. Taken together, these results indicate that exosomal HMGB1 secretion from tumor cells promotes the formation of an immunosuppressive microenvironment and reduces antitumor responses. The forms of HMGB1 and the manner by which HMGB1 is released during different pathological or physiological processes remain unclear, and the HMGB1 function may depend on its forms and its mode of secretion.

Numerous strategies aim to inhibit HMGB1 activity or release. Recombinant HMG Box-A is a specific antagonist of HMGB1 protein [43]. Box-A suppresses mesothelioma cell viability, migration, and growth in vitro [44] and significantly decreases the growth of mesothelioma tumors in a xenograft mice model, thereby extending the survival of mice without side effects [45]. Ethyl pyruvate (EP), a pyruvic acid derivative, is an inexpensive and safe compound with effective antitumor activities [46]. EP has the potential roles in suppressing inflammation related tumor progressions by inhibiting the HMGB1 and corresponding downstream pathways [43, 47]. In vivo, EP could prevent the tumor growth and extend the survival of large B-cell lymphoma (DLBCL) mice model by downregulating the expression of HMGB1 and phosphorylation of ERK1/2 and Src [48]. EP induced cell-cycle arrest and apoptosis of hepatocellular carcinoma tumor cells in vitro and significantly inhibited the pro-tumor inflammation pathway of MC38 in a dose-dependent manner in a colorectal cancer mice model, thereby suppressing tumor growth [49, 50]. Furthermore, EP treatment significantly decreased the release of HMGB1 from DLBCL cells, thus inhibiting tumor cell proliferation in vitro. EP also inhibits the formation and progression of gastric, gallbladder, and prostate cancers by downregulating the release of HMGB1 and preventing activation of corresponding pathways [51–53]. A recent study demonstrated that triptolide significantly inhibited the viability and clonogenic ability of cancer cells by downregulating HMGB1 expression, thereby suppressing breast cancer growth. After treatment with triptolide in vitro, expression of the downstream HMGB1 correlation factor TLR4 and phosphorylated NF-κB p65 was significantly reduced. In vivo, the anti-tumor activity of triptolide also has been confirmed, and triptolide treatment significantly inhibited tumor growth in a BALB-c mice model bearing MDA-MB-231 cells [54]. In addition, HMGB1 promotes the resistance of osteosarcoma to chemotherapy in vitro, and overexpression of miR-22, which targets the 3′ untranslated region (UTR) of HMGB1, inhibits HMGB1 function and reverses the resistance of tumor cells to chemotherapy [55]. These studies reveal the importance of targeting HMGB1 in anti-cancer therapy.

### ANXA2

Annexin A2 (ANXA2), expressed on the surface of mononuclear cells, macrophages, endothelial cells (ECs), and various kinds of cancer cells, is a Ca^2+^-dependent phospholipid-binding protein. ANXA2 plays a crucial role in several types of biological processes, such as autophagy, exocytosis, cell–cell communication, endocytosis, and biochemical activation of plasminogen. A recent study described a novel pathway of ANXA2 extracellular transport that consists of Ca^2+^-dependent exosomal transport. In this pathway, ANXA2 binds to lipid rafts, which transports it through an endocytic pathway that is associated with the intralumenal vesicles of multivesicular endosomes. Then, the multivesicular endosomes containing ANXA2 directly fuse with the plasma membrane and release intralumenal vesicles into the extracellular environment, promoting ANXA2 transfer from the cell to cell. Intracellular levels of Ca^2+^ can influence the combination of ANXA2 and lipid rafts. Stimulation by a Ca^2+^ ionophore induces the binding of ANXA2 to the specialized microregions of the exosome membrane with raft-like characteristics. These observations indicate that the trafficking of ANXA2 is dependent on plasma membrane rafts. Moreover, to escape the endosomal degradation pathway, ANXA2 can be selectively incorporated into the lumenal membranes of the endosomes [56], and interferon-γ (IFN-γ) induces the exosome release of ANXA2 in an amphisome-dependent manner [57]. Treatment of cells with IFN-γ resulted in amphisome colocalization with ANXA2, CD63, and LC3B. This colocalization and subsequent exosome release were dependent on RAB27A, ATG5, and RAB11, indicating that the formation of MVBs and autophagosomes, as well as amphisomes, and their subsequent fusion with the plasma membrane are important to the endosomal release of ANXA2. Moreover, ANXA2 is one of the most highly expressed proteins in exosomes derived from cancer cells, and exosomal ANXA2 triggers the activation of the STAT3, p38, and NF-κB pathways in and the enhanced secretion of TNFα and IL6 from macrophages [58]. In addition, exosomal ANXA2 promotes tPA-dependent angiogenesis, and depletion of exosomal ANXA2 decreases metastasis of breast cancer to the lung and brain [59] (Fig. 1). In summary, the secretion of tumor-derived exosomal ANXA2 depends on intracellular calcium levels and novel autophagy-mediated secretion. Extracellular ANXA2 accelerates angiogenesis and contributes to the formation of an immune microenvironment that promote tumor metastasis.

Targeting ANXA2 also showed potential value in terms of anti-tumor therapy; the monoclonal antibody ch2448, which targets ANXA2, was reported to kill breast and ovarian cancer cells in vivo as well as in vitro via antibody-dependent cell-mediated cytotoxicity (ADCC) and/or antibody-drug conjugate (ADC) routes [60]. In vivo treatment of human embryonic stem cells (hESCs) with ch2448 post-transplantation eliminated circulating undifferentiated cells and prevented or delayed the formation of teratomas [61]. In addition, Ginsenoside compound K exhibits remarkable anti-tumor activity in several types of cancer cells and animal models by inhibiting the nuclear colocalization of and interaction between ANXA2 and the p50 subunit of NF-кB to prevent the activation of NF-кB and the expression of downstream genes [62]. These studies reveal the great research value of ANXA2 in tumor therapy.

### ATP

Adenosine triphosphate (ATP), the most abundant metabolite in the cell, is also a crucial autocrine/paracrine messenger that functions by binding to denatured (P2Y) or ionic (P2X) purinergic receptors [63]. In addition to being involved in purinergic neurotransmission, ATP and its derivatives act as signaling molecules in a variety of cellular processes, such as immune regulation, mucociliary clearance, and vasodilation. Intracellular ATP can be released in response to various cell death-associated and cell stress-associated conditions, such as exposure to cytotoxic agents, hypoxia, plasma membrane damage, and shear stress following mechanical disruption. Various mechanisms regulate the release of stress-induced ATP, including the active exocytosis of ATP-containing vesicles and the secretion of cytoplasmic ATP via pannexin channels, gap junction hemi-channels, and transporters of the ATP-binding cassette family [64]. Autophagy is required for the optimal release of ATP following the application of antineoplastic chemotherapies [65]. In mammalian tumor cells, some of the VAMP7 (V-SNARE)-positive vacuoles colocalize with LC3 at the periphery (focal adhesions) of the cell during starvation. This redistribution of VAMP7-positive structures is dependent on the participation of microtubule proteins, such as the RAB7 effector RILP and the motor protein KIF5, and it is interesting to note that most VAMP7-tagged vesicles contain ATP. Moreover, in starved cells, these structures release nucleotides to the extracellular space by fusing with the plasma membrane [66]. Accumulation of ATP in the extracellular medium can be sensed by purinergic receptor P2Y2, which is important for the recruitment of CD11b + CD11c + Ly6Chigh cells to the tumor microenvironment [67, 68]. Local ATP concentrations can promote the differentiation of CD11b + CD11c + Ly6Chigh cells into mature DCs, which are capable of cross-presenting tumor antigens and promoting subsequent activation of CTL responses [69]. In addition, ATP can bind to P2RX7, another purinergic receptor, to upregulate the IL-1β produced by the NLRP3 inflammasome [70]. Then, this IL-1β can stimulate the activation of CTL responses actuated by γδT17 cells [71]. To resist these immunostimulatory signals, some tumor cells and Tregs overexpress the ectonucleotidases CD39 and CD73 to metabolize ATP to adenosine. Similarly, exosomes derived from cancer cells also express CD39 and CD73, subsequently inhibiting T-cell activation through the adenosine A(2 A) receptor [72] (Fig. 1). Pharmacological blockade of CD39 by a novel sodium polyoxotungstate inhibitor NTPDase stimulated antitumor immunity in several models and reduced tumor cell metastasis [73]. Moreover, treatment of murine tumor models with anti-CD73 antibodies also reveals its ability to inhibit tumor cell metastases and migration [74, 75]. At present, two early-phase clinical trials are aiming to investigate CD73 monoclonal antibodies in cancer treatment. A Phase 1/2a trial designed to assess the safety and efficacy of CD73 antibody MEDI9447 combined with anti-programmed death ligand 1 (PD-L1) therapy in patients with advanced solid tumors is currently recruiting, and interim results have not been disclosed to date (NCT02503774). Moreover, a Phase 2 clinical study investigating the efficacy of anti-CD73 MEDI9447 in combination with other immune checkpoint inhibitors, including MEDI0562, tremelimumab, and durvalumab, in the treatment of relapsed ovarian cancer is currently underway (NCT03267589).

Taken together, in contrast to being directly secreted outside cells, ATP contained in EVs in the autophagy pathway may be protected from being decomposed by the ectonucleotidases and then plays an antitumor role. The data indicate that extracellular ATP contributes to the effectiveness of antitumor immune response, which depends on ATP concentration, the secretion pathway affected, and key metabolic enzymes in the tumor microenvironment. Moreover, targeting CD39 or CD73 seems to be a promising anti-tumor strategy.

### DNA

DNA damage caused by ionizing radiation and topoisomerase inhibitors results in the accumulation of cytoplasmic DNA that contains a large amount of single-stranded DNA (ssDNA) and small amounts of double-stranded DNA (dsDNA). Exosomal DNA (exoDNA) originated in the mitochondria (mtDNA) and nucleus represent the entire genome and are not biased toward a particular DNA structure or sequence [76]. Interestingly, exoDNA was initially discovered as an ssDNA [77]. Later, the major form of exoDNA was found to be dsDNA located at the surface and inside of the vesicle [76]. Compared to that in the exosomes from noncancer cells, the level of exosomal DNA was significantly higher in cancer-derived exosomes [76]. Considering the high instability of the genome and the accumulation of cytoplasmic DNA in cancer cells, it is possible for cancer cells to secrete more exoDNA to prevent aging and death, thus enhancing cancer cell survival [78].

Although exosomes derived from cancer cells contain more DNA, the functional effect of exoDNA uptake remains unclear. Cytoplasmic dsDNA is a DAMP that can stimulate type I IFNs and other cytokines by cGAS/ STING signaling [79–81]. In addition, mitochondrial DNA (mtDNA) can be transferred via tumor-secreted exosomes [82] and be recognized by factors in the cGAS-cGAMP-STING signaling axis [83] (Fig. 3). Dying cells recruit cGAS-STING pathway factors by releasing mtDNA to increase the production of interferon [84]. However, because cGAS cannot bind ssDNA, ssDNA generally induces fewer type I IFNs than are induced by dsDNA [85]. It has been shown that nicked dsDNA can be degraded by Trex1, an exonuclease. However, in cells lacking Trex1, chronic autoinflammatory phenotypes induced by IFN are also associated with the accumulation of ssDNA [86]. After DNA is damaged, these ssDNA can form double-stranded secondary structures, further promoting activated cGAS, and can be degraded by Trex1. Recently, it was shown that ssDNA is also carried by microvesicles to recapitulate genomic aberrations; for example, microvesicle-trafficked ssDNA can amplify the oncogenes (i.e., MYC) of the primary tumor [77]. In the setting of metastatic cancer, higher levels of exosomal dsDNA have been found in aggressive melanoma compared with the levels found in nonmetastatic or low-metastatic melanoma [87]. Prominently, the dsDNA contained in exosomes exhibits the oncogenic mutation status of the respective cancer cell of origin [76], highlighting the fact that exosomal dsDNA can be characterized as a favorable biomarker to detect oncogenic mutations in clinical applications. The role of exoDNA derived from tumors has been mainly studied in cases of radiation treatments or chemotherapy. For example, in mice with BC tumors, immunostimulatory DNA secretion can be induced by treatment with topotecan or by irradiation, triggering an antitumor response by promoting the maturation of DCs and activation of CD8^+^ T cells [88]. Due to the complex functions of exoDNA in regulating the immune responses or physiological process of tumor cells, inoculated engineered exoDNA may exhibit potential value in anticancer therapy. For example, one study described that treatment with exosomal mtDNA, which is derived from cancer-associated fibroblasts, could reverse the drug resistance of hormonal therapy-resistant metastatic breast cancer [89]. All together, these findings illustrate that EVs containing DNA are characterized by the paracrine signals that are initiated by the DNA damage response with adaptive alteration. The form and amount of DNA that is packaged into EVs are unclear. In addition, the mechanisms by which intracellular DNA is secreted and their extracellular functions require more research.

### RNA

Various RNAs are contained in EVs, and many studies have shown that long noncoding RNAs (LncRNA) and microRNAs (miRNAs) are the major species of RNA transported by exosomes; the presence of tRNA, rRNA, and mRNA has also been reported. The species and amount of RNA in EVs depends on the initiation and stress level of the cells [90]. For example, miR-23a-3p is one of the most enriched miRNAs in hepatocellular carcinoma (HCC) cell exosomes during ER stress and can inhibit the expression of PTEN and increase the expression of phosphorylated AKT and PD-L1 in macrophages, which subsequently decreases the production of interleukin-2 and the ratio of CD8^+^ T cells but increases the number of apoptotic T cells [91]. Accumulating evidence ravel the role of exo-miRNA in chemotherapy resistance of tumor cells. exo-miR-1246 expression was significantly associated with the chemosensitivity of ovarian cancer (OC). Overexpression of exo-miR-1246 could promote chemotherapy resistance in tumor cells. Treatment with a miR-1246 inhibitor combined with chemotherapy reduced tumor burden in vivo [92]. Furthermore, EVs-modified PEI/siRNA complexes against miR-1246 exhibited marked suppression of tumor growth in a PC3 prostate carcinoma mice model, suggesting the promising of targeting miR-1246 in cancer therapy [93]. Although miRNAs contained in exosomes have been the most extensively studied, the functions of other exosomal RNAs remain unclear. Recent studies have described the mechanism of RNA-DAMP transfer by cancer-derived exosomes. In normal physiological processes, RN7SL1 is generally bound with SRP9/14, which shields it in the fibroblast cytoplasm. However, the fibroblasts in BC can deploy these unshielded RN7SL1 proteins in exosomes through the activated Notch-Myc pathway induced by the tumor cells, and this unshielded RN7SL1 can induce an inflammatory response through activated RIG-I that is transported to immune cells and can promote the growth and invasion of tumors when it is transported to BC cells [8] (Fig. 3). Additionally, both anthracycline and oxaliplatin can promote type I IFN production in malignant cells through the stimulation of endosomal TLR3, although the precise anthracycline-elicited ligand(s) that stimulate TLR3 remain to be verified, but it can be posited that this cascade of cancer promoting action is the result of a maladjusted structure of self RNA that is released from dying or stressed cancer cells. Indeed, it has been shown that double-stranded RNA molecules generated through DNA-damaging agents can trigger the secretion of TLR3-dependent cytokines [94]. Additionally, anthracycline-induced type I IFN signaling can increase the production of a potent chemoattractant, CXCL10, which recruits T cells into the tumor microenvironment. Interestingly, RNA transportation mediated by EVs also promotes the elimination of tumor suppressive molecules from cancer cells. In breast cancer, compared to oncogenic miRNAs, tumor-suppressive miRNAs were mostly packaged into EVs, and this miRNA rebalancing mechanism mediated by EVs favors protumorigenicity, which promotes the progression of primary tumor [95]. In summary, there are multiple types of RNAs that invariably involve immunogenic stress-driven microenvironmental alterations. The possible branches that specifically evolve to sort and link RNA to intracellular stress responses require further investigation.

### HSPs

Heat shock proteins (HSPs) found in all eukaryotes and prokaryotes are highly conserved proteins and are considered pivotal markers of the cellular stress response to various stressful stimuli [96]. These stress responses can improve the cell capacity for coping with accumulated misfolded proteins. The Hsp70 family is the most conserved and best studied class in all the HSP subfamilies [97]. And HSP70 was found as a native tumor antigen in exosomes [98, 99], that was extracted from a variety of stressed tumor cells, including melanoma cells, Lewis lung carcinoma, carcinoembryonic antigen (CEA)-positive tumor cells, and lymphoma cells [100, 101]. In addition, increased release of HSPs (HSP90, HSP70, and HSP60) contained in exosomes was found for human hepatocellular carcinoma cells after treatment with resistant anticancer drugs. Recently, exosomal Hsp72 (eHsp72) was described as a chaperokine that has the function of both a chaperone and a cytokine [102]. APCs stimulated with eHsp70 can trigger specific signal transduction pathways that results in an immune response that profoundly increases the release of cytokines, such as IL-12, IL-6, IL-1β, and TNF-α [103, 104], as well as chemokines, including RANTES, MCP-1, and MIP-1 [105]. Additionally, eHsp72 can induce the maturation of DC and NK cell migration by increasing the expression of CD86, CD83, CD40, and MHC class II molecules on the DC surface [106, 107] (Fig. 2). However, these tumor exosomes induce relatively weak antitumor immune responses and are likely to induce tolerance. Therefore, these strategies are limited to mouse model studies and in vitro observations. Thus, it is clear that EV-derived HSPs, in response to different cellular stresses, contribute to the communication between tumor cells and the local microenvironment. Notably, targeting these HSPs has been recognized as an attractive strategy to sensitize various cancers to chemo- and radiotherapy. HSP27 inhibitors, such as RP101 and quercetin, could enhance the anticancer therapeutic effects in various cell lines including oral cancer and glioblastoma [108, 109]. Moreover, NW457, an inhibitor of HSP90, could induce the apoptosis of conditional reprogramming cells when combined with radiotherapy [110]. In addition, Ganetespib as the inhibitor of HSP90 has also been described as a radiosensitizer in pancreatic ductal adenocarcinoma by modulating the STAT3, HIF-1α as well as AKT-driven pathways [111]. Interestingly, different stress responses may result in diverse HSPs being packaged into EVs, which may be associated with the subtype of HSPs in the specific organelles, thereby targeting HSPs or inoculating engineered exosomes may have potential value in antitumor therapies.

### Lipids

The profiling of lipids in the composition of EVs has been extensive. Sphingomyelins, ganglioside GM3, cholesterol, and phospholipids have all been found to be enriched in vesicles derived from wild-type cells [112]. Importantly, ceramide, which is classified as a sphingolipid, plays a crucial role in the formation and/or secretion of exosomes [113], and the formation of ceramides is mediated by a variety of lipid-modifying enzymes, such as sphingomyelinase (SMase). It has been reported that knocking down SMase has often inhibits EV release [114]. Two major ceramide species, C24:1 and C18:0 ceramide, are contained in exosomes, and vesicles isolated from intracellular membranes also contain C16:0 ceramide [82]. It has been reported that, in response to stress stimuli and several extracellular agents, ceramides were found to accumulate in various cancer cells [115]. In addition, the deregulation of ceramides of specific chain lengths, which are generated by six different ceramide synthases (CerSs), contributes to chemoresistance in several cancer types [115]. In addition, ceramides play a pivotal role in T-cell biology [116]. A recent study showed that exosomes derived from tumors can be laced with alpha-galactosylceramide, which enhances the activation of T cells by dendritic cells, providing a promising strategy against cancer cells via inoculated engineered exosomes [76] (Fig. 2). Based on this observation, the immunoregulatory function of ceramides in EVs may rely not only on its enrichment but also on its composition and proportion.

## Conclusion and perspective

In summary, we have discussed immunogenic stress in cancer mainly from the perspective of extracellular vesicles. Tumors thrive under adverse conditions, such as nutrition deficiency, immune destruction, and anticancer treatment, by modulating their adaptive capacity via several stress responses. In addition, stress-induced EVs could harness the intrinsic ability of the host immune system to recognize and eliminate tumor cells, representing a promising therapeutic strategy. In contrast, recent studies have uncovered an opposite mechanism by which EVs with abnormal contents induce immune cell function remodeling and cause immunosuppression that promotes malignant progression. Therefore, the interplay between the immunomodulatory and stress response is important for anticancer therapy.

The immunogenic stress responses are connected to a complex network of signals that link individual cells to the host organism upon malignant cells reactions to potentially harmful perturbations. The potential cellular reaction during immunogenic stress mainly constitutes activated autophagy, ER stress reactions, and DNA damage response. Upon external stimuli, such as starvation or anticancer drugs, the immunogenicity of cancer cells undergoing immunogenic stress responses appears to be remarkably altered. This effect could facilitate the elicitation of antitumor immunity. Globally, these immunogenic stress responses affect a large spectrum of processes from the local immune-inflammatory response to systemic metabolic homeostasis, and targeting immunogenic stress may stimulate tumor immunogenicity to improve the efficiency of cancer immunotherapy.

It is tempting to speculate on the mechanisms bridging cellular stress responses to the microenvironment and macroenvironment. In support of endeavor, EVs are regarded as messengers that initiate communication between stressed tumor cells and the immune system; however, several issues remain outstanding. Primarily, a comprehensive understanding of EV biology, particularly the mechanism by which specific immunogenic proteins, DNA and RNA are packaged into EVs, needs to be established. Because tumor-originated EVs contain specific mediators, deciphering how they are selected by malignant cells may reveal targets for therapy. The selective cargoes for EV transport as induced by immunogenic stress remain sheltered within cells under adverse conditions, where they are internalized by immune cells. The inherent characteristics of the EV contents and the targeted receiver of the immune cells determine whether the EVs are immunosuppressive or immunostimulatory.

Acquired resistance to monotherapies is one of the major obstacles in combating cancers and remains a field of active investigation. Therefore, combined treatment is crucial to overcoming therapeutic resistance. It has been well established that the EV-targeted reactions elicited by cellular stress have major implications for anticancer treatment, and EV-targeted therapy is a novel approach for augmenting the potency of standard therapies and anticancer immunotherapies.
